# Comparison of placement characteristics using two intraosseous devices in canine and feline cadavers by novice users

**DOI:** 10.3389/fvets.2023.1196284

**Published:** 2023-07-20

**Authors:** Olivia C. Uzan, Liz S. Guieu, Kelly E. Hall, Claire D. Tucker, Tracy L. Webb, Julie Dunn, Julien Guillaumin

**Affiliations:** ^1^Department of Clinical Sciences, Colorado State University, Fort Collins, CO, United States; ^2^Medical Center of the Rockies, University of Colorado Health, Loveland, CO, United States

**Keywords:** intraosseous, dog, cat, bone, IO catheter, EZIO, SAMIO

## Abstract

**Introduction:**

Intraosseous (IO) catheterization enables rapid access to systemic circulation in critical patients. A battery-powered IO device (BPIO) utilized in veterinary practice is reliable in facilitating IO catheter placement. A new spring-powered IO device (SPIO) has been developed for people but has not been tested in veterinary patients. The goal of our study was to compare placement characteristics and flow rates achieved with the BPIO compared to the SPIO in animals when operated by novice users.

**Methods:**

Six veterinary students performed 72 catheterizations in the humeri and tibias of 12 dog and 6 cat cadavers. The user, cadaver, device, and site of placement were randomized. Flow rates were determined by three-minute infusions.

**Results:**

In dogs, overall success rates (50% BPIO, 46% SPIO; *p* = 0.775) and flow rates based on location were similar between devices. Successful placement was faster on average with the BPIO (34.4 s for BPIO and 55.0 s for SPIO, *p* = 0.0392). However, time to successful placement between devices was not statistically significant based on location (humerus: 34.7 s for BPIO and 43.1 s for SPIO, *p* = 0.3329; tibia: 33.3 s for BPIO and 132.6 s for SPIO, *p* = 0.1153). In cats, success rates were similar between devices (16.7% for BPIO and 16.7% for SPIO, *p* = 1.000), but limited successful placements prevented further analysis.

**Discussion:**

This is the first study to examine the use of the SPIO in animals, providing preliminary data for future IO studies and potential applications for training in the clinical setting.

## Introduction

Delivery of lifesaving treatment can be delayed in dogs, cats, or people experiencing cardiovascular collapse due to repeated failed attempts at placing intravenous catheters (1–5). Placement of an intraosseous (IO) catheter allows rapid access to systemic circulation and is an option for the effective delivery of emergency drugs and fluid therapy (2–7). Complications are rare but have been described in human patients to include, but are not limited to, cutaneous bullae, traumatic fractures, osteomyelitis, and further complications from extravasated fluids such as compartment syndrome and tissue necrosis (8). Hand-held insertion devices have been developed to facilitate the placement of IO catheters (2–6, 9–11). However, affordability, limited reusability, knowledge of the existence of the device, and lack of training might make IO catheterization an under-considered option to access peripheral circulation by veterinary practitioners.

**Figure 1:**
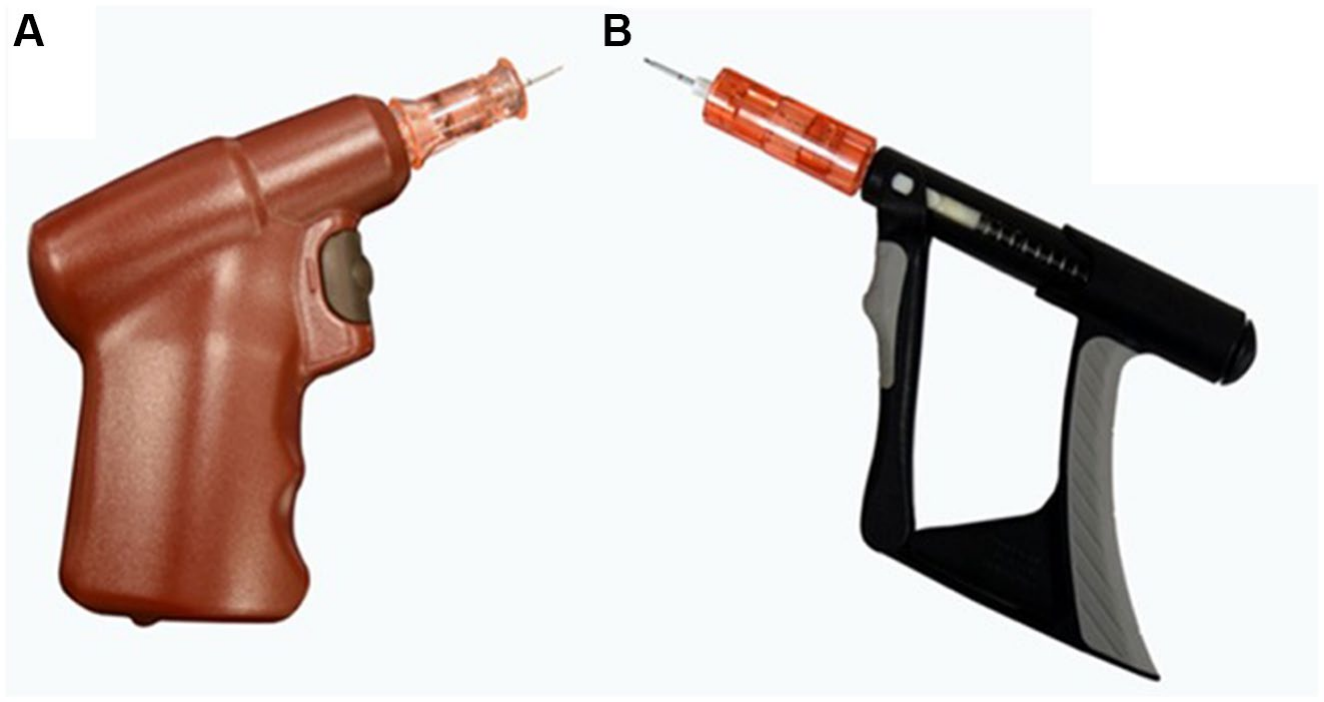
**(A)** Battery-powered intraosseous (BPIO) device driver and catheter needle. The catheter is attached to the device, and the button is compressed to drill the needle to place the catheter. **(B)** Spring-powered intraosseous (SPIO) device driver and catheter needle. Once the catheter has been loaded on the driver, repeated actuation of the trigger creates a rotational spin of the needle assembly and allows a controlled IO catheter placement.

A battery-powered IO device^1^ (BPIO) (Figure 1A) is commonly utilized by emergency veterinarians. It is automatically driven, weighs approximately 140 grams, and has been shown to be reliable in facilitating IO catheter placement and fluid administration in people and animals (4, 5, 9, 12). The battery is non-rechargeable, which limits device lifespan, and is only compatible with brand-specific, single-use catheters (12).

A spring-powered IO device^2^ (SPIO) (Figure 1B) has been developed for use in people. Compared to the BPIO, where a button is pushed to drill in the needle of the catheter^3^, the SPIO functions by continuously actuating (i.e., manually compressing) the trigger^4^. This creates a rotational spin of the catheter needle and makes IO catheter placement possible. Since the spring-powered device does not rely on a battery, it has a longer lifespan (10,000 uses reported in humans), can be submerged for disinfection, and is half the weight of the BPIO. The catheter adapter also makes the SPIO compatible with catheters from other manufacturers (13). To the authors’ knowledge, there is a lack of published data about use of the SPIO in people and no animal studies have investigated the time for placement, attempt number, or success rate of IO catheter placement using the SPIO.

The objective of this pilot study was to compare placement characteristics and isotonic crystalloid fluid flow rates when IO catheters were placed using the BPIO and SPIO by novice users in canine and feline cadavers. We hypothesize that the BPIO will allow for faster successful placement of the IO catheter compared to SPIO but that other characteristics will be similar between devices.

## Methods

A crossover study was designed and conducted at the Translational Medicine Institute at Colorado State University on June 20th, 2022.

### Cadaver subjects

Due to the lack of available data to calculate a sample size, a convenience sample of 12 canine cadavers and 6 feline cadavers without any gross pathology of the bones were selected for IO catheter placement from university-approved vendors^5^^,^^6^. The cadavers were maintained frozen and went through one freeze–thaw cycle. Thawing time was approximately 14 days in a cooler prior to use for our study. Twelve mixed breed dog cadavers were used; 83.3% were male and 16.7% female, with a median body weight of 25.9 kg (range 21.3–34.9). All the dogs were visibly well-muscled, notably around the lateral proximal humerus. Cadaver sizes were based on availability. Their ages were unknown. Six female cat cadavers were used, with unknown ages and a median body weight of 2.75 kg (range 1.9–3.5). Five were domestic shorthairs, and one was a domestic long hair.

### Study participants

Six novice users, defined as veterinary students having no previous experience with IO catheterization, were recruited by blast e-mail to second-, third-, and fourth-year veterinary students at Colorado State University for participation in the study. All novice users were veterinary students that identified as female. Out of these six study participants, 66.7% completed their third year and 33.3% completed their first year. None of the participants had prior experience to placing IO catheters.

### Study participant training

A video tutorial showing how to place each device in the tibia and humerus of a canine cadaver was sent to study participants for review 3 days before the experimental day. The palpable landmarks discussed in the video were the acromion, humeral head, and lateral aspect of the humerus, or the stifle joint, tibial tuberosity, and 1.5 inches distal to the tibial plateau for IO catheter placement in the proximal lateral humerus and the proximal medial tibia, respectively.

### Anatomical sites

The animals were prepared by shaving all IO insertion sites (proximal lateral humerus and proximal medial tibia) on both sides of each cadaver. The landmarks were palpated, and a stab skin incision was performed with a #10 scalpel blade on each animal at the expected IO catheter insertion site by one of the investigators (LG). All cadavers were initially positioned in left lateral recumbency so the first lateral humerus placement would always occur on the right forelimb and the first medial tibia placement would be on the left hindlimb. The cadavers were labeled with a number (i.e., 1 to 12 for dogs and 1 to 6 for cats) that was randomly assigned as they were prepared.

### Experiment

Two BPIO and two SPIO devices were used at random for the entire study. Single-use, device-specific 15-gauge catheter needles were used. Two types of catheter needle lengths were used for each cadaver species to account for species anatomical size differences (25 mm long needles for dogs and 15 mm for cats). The term “catheter” will be used to also include the IO catheter needle throughout the manuscript moving forward. The study occurred in 6 rounds. For each round the study participant attempted IO catheter placement using one IO device in one anatomic location in one cadaver followed by attempt to place an IO catheter using the other IO device in the contralateral anatomic location. Randomization of study participant, first device used (i.e., BPIO or SPIO), species (i.e., dog or cat), cadaver number, and site of placement (i.e., tibia *vs* humerus) was predetermined by block-of-6 randomization. For example, Study Participant 3 was randomized to attempt placement of IO catheter using the BPIO in the left tibia of Dog 6. Study Participant 3 would then attempt placement of IO catheter using the SPIO in the right tibia of Dog 6. Therefore, each participant placed a total of 12 IO catheters, 6 using the BPIO and 6 using the SPIO. Only one participant was allowed in the experimental room at a time so that they would be blinded to each other’s performance.

Prior to IO catheter placement, participants were allowed to identify bony landmarks. They stood next to the animal with the unloaded IO device and appropriately sized catheter, unpackaged and uncapped, both placed on a nearby stand. Placement attempt time started when the users verbalized “*I am ready*” and ended once they had detached the IO device from the catheter, were satisfied by their IO placement, and verbalized “*I am done.*” Participants were allowed a maximum of three placement attempts, for a maximum of 5 min (300 s) for each site.

The participant was dismissed from the room, and successful placement of the IO catheter was determined by agreement between two of the investigators (LG, CT). An unsuccessful placement means that the catheter either failed to be placed deep enough through cortical bone or it was placed too deep where it penetrated through the bony cortex on the other side. The catheter could be seated subcutaneously, intramuscularly, or halfway through the cortex. In successfully placed IO catheters that penetrated the cortex and seated in the medullary cavity, flow rate through the IO catheter was then evaluated. A 1 L bag of 0.9% NaCl^7^ was connected to an infusion set^8^ and directly connected to the IO catheter. The initial fluid bag and infusion set was weighed^9^ in triplicate before administration, and the weight was averaged. The bag was placed in a pressure sleeve^10^ and hung using an intravenous (IV) fluid pole. The height of the pole was adjusted so that the top of the fluid bag would stand 90 cm above the catheter insertion site while in the sleeve. The pressure sleeve was inflated to 300 mmHg, and pressure was maintained at 300 mmHg during fluid infusion. After the fluid line was connected to the IO catheter, fluids were administered for 3 min. At the end of the infusion, the final fluid bag and infusion set were weighed in triplicate and averaged. The catheter was discarded, and the cadaver was turned onto right lateral recumbency, and the same study participant was escorted back into the experimental room. The participant was then asked to place another IO catheter in the contralateral limb of the same cadaver using the other IO device.

Boney landmarks, angles of insertion of both anatomic locations, finger position and speed of repeated compressions for the SPIO, catheter depth, and the sensation felt once the catheter was properly seeded through cortical bone were reviewed in person between the 1st and the 2nd round of novice user placements by two of the investigators (LG, JG).

### Data recorded

**Figure 2:**
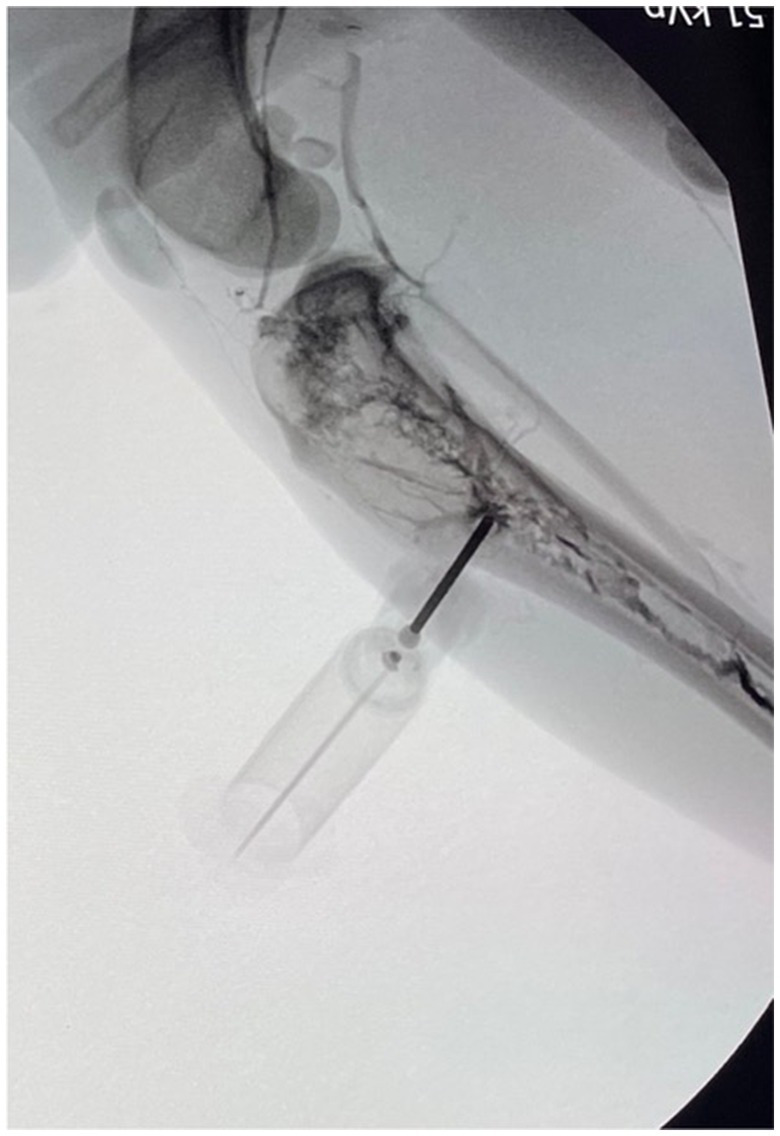
Fluoroscopic image following intramedullary injection of ioxehol in a successfully placed intraosseous catheter in a canine tibia using a spring-powered device.

Data recorded included species, breed, sex, and body weight of the cadaver, study participant number, IO device used, IO insertion location, success of placement, successful placement time, number of placement attempts, and fluid rate. A placement attempt was defined as the user placing the IO catheter within bone with a new attempt marked by removal of the catheter from the insertion site and redirection. An attempt was defined as successful if the catheter was firmly seeded in the bone marrow, and bone marrow could be retrieved by applying negative pressure on a 10 mL syringe. If no bone marrow could be retrieved but the catheter was firmly seeded through the cortical bone, an attempt was defined as successful if there was no resistance to the injection of 10 mL of tap water through the IO catheter with no observed signs of extravasation or if a contrast agent could be visualized in the medullary cavity using fluoroscopy^11^ (Figure 2) after injection of 3 mL of undiluted iohexol^12^ through the IO catheter. The catheter also had to remain patent through fluid infusion to be considered successful; any obvious signs of extravasation re-classified the attempt as unsuccessful, and fluid infusion was aborted.

Flow rates were calculated in mL/kg/min by subtracting the difference in the pre- and post-infusion fluid bag weights (assuming 1 g was equivalent to 1 mL), dividing the obtained volume by the animal’s body weight, and accounting for the three-minute infusion period. Flow rates were reported in mL/kg/min and converted to L/h for clinical interpretations.

### Data analysis

Statistical analyses were performed by an independent biostatistician not involved in the study design and blinded to device evaluated. The continuous data were evaluated for normality assumption using Shapiro–Wilk statistics. If normality was met, data were presented using mean ± standard deviation and a Student t-test was used to compare the two groups. If normality was not met, data were presented using median (range) and a Wilcoxon two-sample test was used to compare the two groups. For categorical variables, differences were tested using a Chi-square test or Fisher’s Exact Test if one of the cells were less than 5. value of *p* of 0.05 was used to determine statistical significance. A commercially available statistical software was used to analyze all data^13^.

## Results

### Canine experiment

All six study participants attempted to place an IO catheter using one of the two IO devices with a 25 mm long IO catheter in the predetermined anatomical location.

#### Successful placement rate in dogs

**Figure 3:**
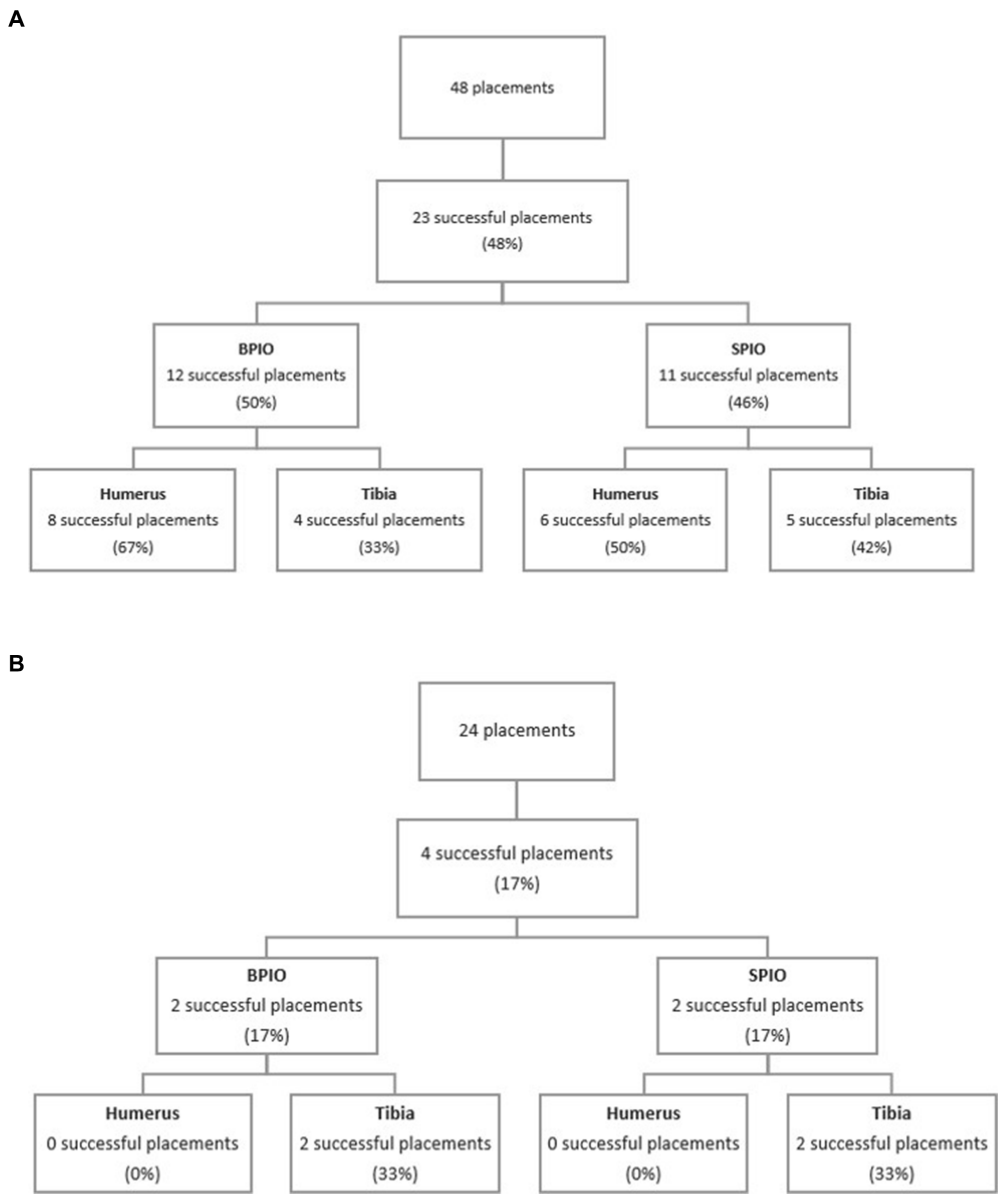
**(A)** Flow diagram of successful placements between intraosseous devices and bones in dogs. **(B)** Flow diagram of successful placements between intraosseous devices and bones in cats. BPIO, Battery-powered intraosseous device; SPIO, spring-powered intraosseous device.

When attempts using both devices were combined, the overall success rate for IO placement was 47.9%. Similar overall rates of successful placements were observed between devices (50% for BPIO and 45.8% for SPIO, *p* = 0.7726) (Figure 3A). Successful placement was comparable between bones when both devices were combined (humerus 58.3%, tibia 37.5%; *p* = 0.2429). When comparing devices used in the humerus, similar catheter success rates were found (BPIO 66.7%, SPIO 50.0%; *p* = 0.6802). There was also no difference between device success rates in the tibia (BPIO 33.3%, SPIO 41.7%; *p* = 1.000) (Table 1).

**Table 1:** Placement characteristics and flow rates recorded using the BPIO and SPIO in the humerus and tibia of canine cadavers.

	BPIO	SPIO	Value of *p*
Number of successful placements: humerus	8	6	NA
Number of successful placements: tibia	4	5	NA
Success rate (%)	50	45.8	0.7726
Success rate in humerus (%)	66.7	50.0	0.6802
Success rate in tibia (%)	33.3	41.7	1.0000
Placement time in humerus^†^ (sec)	34.7 (26.8–218.0)	43.1 (30.2–77.2)	0.3329
Placement time in tibia^†^ (sec)	33.3 ± 9.3	132.6 ± 108.7	0.1153
Flow rate in humerus (L/h)	2.62 ± 1.84	3.79 ± 2.75	0.3557
Flow rate in tibia (L/h)	0.81 ± 0.87	1.28 ± 0.81	0.4335

BPIO, battery-powered intraosseous device; SPIO, spring-powered intraosseous device; NA, not applicable; ^†^Only successful placements reported.

#### Successful placement times in dogs

The median time for successful placement was faster with the BPIO (34.4 s, range 21.0–218.0) as compared to the SPIO (55.0 s, range 30.22–300.0) (*p* = 0.0392). When successful placement times between devices were analyzed by location, the median time for successful placement in the humerus was similar between devices (BPIO 34.7 s (26.8–218.0), SPIO 43.1 s (30.2–77.2); *p* = 0.3329). Although the average time for successful placement in the tibia was lower for the BPIO (33.3 ± 9.3 s) compared to the SPIO (132.6 ± 108.7 s), it did not reach statistical significance (*p* = 0.1153) (Table 1).

#### Number of attempts in dogs

Of the eight successful placements using the BPIO in the humerus, successful placements were made on the first attempt in 6 dogs and on the second attempt in two dogs. All of the six successful placements using the SPIO in the humerus were on the first attempt. For the tibia, all four successful placements using the BPIO were made on the first attempt. Successful placements were made on the first attempt in three dogs, second in two dogs, and third in one dog using the SPIO in the tibia (Table 1).

#### Fluid rate for successfully placed IO catheter in dogs

When both devices were combined, the average fluid rate was higher in the humerus (1.91 ± 1.36 mL/kg/min, 3.12 ± 2.26 L/h) compared to the tibia (0.65 ± 0.48 mL/kg/min, 1.07 ± 0.82 L/h) (*p* = 0.0168). No difference in average flow rate was observed between devices in the humerus (BPIO: 1.69 ± 1.29 mL/kg/min (2.62 ± 1.84 L/h), SPIO: 2.20 ± 1.51 mL/kg/min (3.79 ± 2.75 L/h); *p* = 0.3557). Similarly, the mean flow rate with the BPIO was similar to the SPIO in the tibia (0.55 ± 0.56 mL/kg/min (0.81 ± 0.87 L/h) and 0.74 ± 0.44 mL/kg/min (1.28 ± 0.81 L/h), respectively; *p* = 0.4335) (Table 1).

### Feline experiment

All six study participants attempted to place an IO catheter using one of the two IO devices with a 15 mm long IO catheter in the predetermined anatomical location, except in one instance where a 25 mm long catheter was selected for IO placement with the BPIO in the tibia. Intention-to-treat statistical analysis was performed.

#### Successful placement rate in cats

One successful placement in a cat humerus with the SPIO was reclassified as “unsuccessful” when extravasation was noted upon rapid fluid infusion for calculation of the fluid rate. This placement was considered unsuccessful for statistical analysis and not used for fluid rate calculation.

When both devices were combined, the overall success rate was 16.7% with only four successful placements out of 24 attempts (Table 2). Two IO catheters were successfully placed with the SPIO (16.7%), and two were placed using the BPIO (16.7%) (*p* = 1.000). The humerus had a 0% success rate while the tibia had a 33.3% success rate (Figure 3B). Due to the low successful placement rate, no further statistical comparison regarding successful placement between devices or bones was performed.

**Table 2:** Placement characteristics and flow rates recorded using the BPIO and SPIO in the tibia of feline cadavers.

	BPIO	SPIO	Value of *p*
Number of successful placements	2	2	NA
Success rate (%)	33.3	33.3	NA
Placement time (sec)^†^	21.3	32.9	NA
Flow rate (L/h)	1.81 ± 0.41	1.34 ± 0.02	0.2408

BPIO, battery-powered intraosseous device; SPIO, spring-powered intraosseous device. NA, not applicable. ^†^Only successful placements reported.

Due to study randomization, one user performed 67% (i.e., eight out of 12 placements for each device) of the IO catheter placement in the humerus. Subjectively, many users appeared to go through the entire tibia bone in the cat cadavers, regardless of the device.

#### Successful placement times for cats

Successful IO placement in the tibia with the BPIO (*n* = 2) took a median of 21.3 s (range 19.6–22.9) compared to 32.8 s (range 25.7–39.9) for the SPIO (*n* = 2) (Table 2).

#### Number of attempts in cats

All BPIO successfully placed IO catheters were made on the first attempt (*n* = 2) in the tibia. One SPIO catheter was placed on the first attempt while the other one was placed on the second attempt in the tibia (Table 2).

#### Fluid rate for successfully placed IO catheter in cats

The median flow rate for the four successfully placed catheters in the tibia was 10.71 mL/kg/min (range 7.8–18.4) or 1.4 L/h (range 1.3–2.1). No difference was observed between devices, with a median of 14.10 mL/kg/min (range 9.8–18.4) or 1.8 L/h (range 1.5–2.1) for the BPIO and 9.71 mL/kg/min (range 7.8–11.6) or 1.3 L/h (range 1.3–1.3) for the SPIO (*p* = 0.3557) (Table 2).

## Discussion

This is the first published study to evaluate the use of the SPIO in veterinary or human medicine. We showed comparable placement characteristics and flow rates for both devices placed in canine and feline cadavers by novice users, though there was an overall low success rate in dogs and worse in cats. Although not statistically significant, placement of IO catheters in the canine tibia were found to be 100 s faster when the BPIO was used compared to the SPIO; the time difference could be clinically relevant in some critically ill patients and deserves further investigation. The observed differences in successful placement times between the canine tibia and the humerus using the SPIO (i.e., 43 vs. 132 s) were expected. Although the specific anatomic locations where IO devices were placed in both bones was not investigated in our study design, it has been showed that the tibia has a thicker proximal cortex compared to the humerus in dogs (14). The increased effort required when using the SPIO device would therefore require increased time to pass through the thicker tibial cortex. In feline cadavers, only four IO catheters were successfully placed in the tibia, which limited statistical comparison of device performance.

The overall successful IO placement rate in canine cadavers by novice users was approximately 50%, with no differences noted between devices. This contrasts with success rates of 87 to 100% previously reported in veterinary medicine (3, 4). The Allukian et al. study using the same BPIO device demonstrated an 87% success rate as compared to 67% in our study in the humerus location (3). The level of clinical experience of the users in the Allukian et al. study did not appear to affect the success rate for IO placement: a veterinary student in their final year successfully placed IO catheters using the BPIO in 83% of cases similar to performance of a veterinary technician specialist (83%), first year emergency and critical care resident (100%), and diplomate of the American College of Veterinary Emergency and Critical Care (83%) (3). Although all users in the Allukian et al. study were generally more clinically experienced than the novice users in our study, the users in these 2 studies were inexperienced with use of the IO devices. Notably, the current study used relatively homogenous medium-large sized cadavers weighing 21–35 kgs, which were available at the study time, while a wide range of breeds weighing 6.2–40 kgs were used in the Allukian et al. study (3). Smaller sized cadavers could have influenced the canine success rate to mimic that which was found for cats. The larger number of participants and difference in training (i.e., video training compared to onsite training), could also have played a role in variable success rates between studies.

Another study investigated success of placement of IO catheters in the humerus and tibia of canine cadavers by two emergency room veterinarians with one to 3 years of clinical experience using the same BPIO device as used in the current study (4). The study participants received in-person training using canine cadavers and were required to successfully demonstrate placement in all locations under supervision of an emergency and critical care specialist, who had extensive experience in IO catheter placement using a BPIO, prior to starting the study. A 100% success rate in the humerus and an 82% success rate in the tibia were reported compared to 67% in the humerus and 33% in the tibia in the current study. As mentioned above, differences in overall clinical experience (i.e., veterinary students without clinical experience versus emergency veterinarian with up to 3 years of experience) or the training provided (i.e., videos versus hands-on and in-person training) might explain differences in success rates observed. Accordingly, live demonstration and immediate feedback should be considered when training veterinarians in IO catheter placement. We attribute the low success rates of IO catheterization with the canine cadavers to the cadaver muscle mass, lack of user experience, and chosen method of training which excluded immediate feedback.

In cats, the overall successful placement rate of the IO catheter was much lower than that in dogs as only 16.7% of all attempts were successful. None of the placements were successful in the humerus, while 16.7% of placement were equally successful in the tibia regardless of the device used. Bukoski et al. reported a 96% success rate when IO catheters were placed in the humeri and tibias of feline cadavers using the same BPIO used in the current study by two veterinarians with clinical experience but minimal experience with IO devices (10). Although clinical training was not reported, each participant placed 36 IO catheters in the cats and the increased repetition likely contributed to the higher observed success rate in cats The randomization scheme for the current study resulted in an uneven distribution of user attempts, with one participant placing most of the feline catheters while the rest of the participants placed a minimum of two. The lack of repetition for the other users may have contributed to the lower success rate. The lack of success in humeral IO placement in cats in the current study is somewhat surprising. Differences in users’ experience and training, as well as the cats’ bone density, cortical thickness, or medulla thickness may have played a role. Although a previous study showed that the tibial bone density is similar in cats when compared to dogs, data regarding differences between humeral and tibial bone density or thickness of the cortex and medulla are lacking in cats (15). We attribute the low success rates of IO catheterization with the feline cadavers to the lack of repetition, lack of users’ experience, and chosen method of training which excluded immediate feedback.

Successful placement time for canine humeral IO catheters was not different between devices, but the placement time for canine tibial IO catheters was four times less for the BPIO compared to the SPIO. Although this difference was not statistically significant, it is likely clinically relevant and may suggest the presence of a type II error. In comparing IO placement times with previous studies, Hafner et al. found the BPIO to be faster than a traditional manual IO catheter when used by resident emergency room physicians in anesthetized swine tibias (11). Their median BPIO insertion time was 3.66 s, with the recording time beginning with skin puncture, whereas the current study included time to assemble the catheter onto the device. Lange et al. reported a BPIO median tibial placement time of under 25 s in canine cadavers when used by inexperienced emergency room veterinarians, consistent with the 33 s in the current study (4). The successful humeral placement times with both IO devices (i.e., 35–43 s) in the current study are similar to another study using operators with varying veterinary experience levels who recorded a median humeral placement time of 55.4 s using the BPIO, which included time to gather materials and prepare the device (3). We elected to have an investigator perform the skin incisions to remove an extra variable unrelated to the study objective and focus on the IO assembly and placement time by itself.

Minimal comparison is possible with successful IO placement times in cats due to the low number of successful placements overall and the lack of successful humeral catheterizations in our study. For tibial IO placements in cats, the current study had a median time for successful placement of 21.3 s compared to 72.0 s reported by Bukowski et al. (10). Direct comparisons are challenging as animal positioning, cursory hair clipping, and skin preparation with three scrub cycles were included in the timing in that study (10).

Our study found that novice users required a second attempt in 25% of the canine humeral placements using the BPIO. This contrasts with a previous study where users did not exceed a single placement attempt in the humerus using the same BPIO (4). As previously discussed, training differences between the two studies may explain this finding (4). However, novice users in the current study did not exceed more than one attempt in the dog humerus when using the SPIO. The spring-powered mechanism and/or the handle design of the SPIO may make it to easier navigate the insertion site in this well-muscled area. Tibial placements with the BPIO did not require more than one attempt, which is consistent with a study describing emergency physicians using the BPIO in anesthetized swine (11). Alternately, 50% of successful placements in the canine tibia using the SPIO required more than one attempt, which is likely related to increased cortical bone thickness in the canine tibia compared to the humerus (14).

None of the successful cat tibial placements with the BPIO required more than one attempt, and an extra attempt was needed in one case of tibial placement with the SPIO in the current study. Published studies on IO use in cats are limited to use for comparison. One available study found a greater ease of placement using the BPIO compared to two other IO devices as defined by a vascular access scoring system and did not state attempt number (10).

The current study confirms previous findings that pressure infusion rates are higher in the humerus compared to the tibia (4, 9). Although using a more homogeneous dog size range, infusion rates expressed in mL/kg/min in this study are almost identical to a previous study with a similar experimental design (4). When location was considered individually, no differences in infusion rate were found between the two devices. Limited additional information is available comparing infusion flow rates between devices, and none using the specific SPIO used in the current study. Sørgjerd et al. compared the BPIO used in this study with a semi-automated IO device designed for sternal use in people and found that 35% of users experienced poor flow with the BPIO compared to 0% with the semi-automated IO device (16). It should be noted that the semi-automated IO device has three catheters compared to one catheter in the BPIO (16).

The current study has several limitations. To mimic the possibility of these devices being available for untrained users such as general practitioners, we elected to have novice users trained using videos as our study participants. These novice users selected were veterinary students rather than ER and ECC veterinarians from the institution because the latter would have had experience placing the BPIO, which would have introduced a biais. User experience, live training with immediate feedback, and repetition may impact placement and timing results when using these devices. Clinical application regarding isotonic crystalloid infusion rates in live animals must be taken into consideration when interpreting these results, as our study was done in cadavers. However, infusion rates may depend more upon the device used, anatomic insertion site selected, type of medication or fluid being infused, and other features of the infusion kit, than the forces of the circulation system in live dogs and cats (17). The study randomization protocol resulted in one user performing 8 of the 12 humeral placements, which may have skewed the results due to that individual’s performance. A larger participant number may provide variable outcomes and overcome errors associated with a small sample size. Success in the feline cadavers was limited and did not allow us to draw meaningful conclusions regarding the performance of the devices. Additional studies in cats with more experienced users, as well as a different training method, to evaluate placement characteristics using both devices are needed. Finally, it is unclear how the findings from this study using cadavers translate to clinical dogs and cats. We suspect that novice users may have an even lower successful placement rate in a high stress clinical environment, which underlines the need for proper training.

## Conclusion

A spring-powered IO placement device offers a potential alternative and more sustainable option as compared to a battery-powered device for veterinary and human medical use. Our study suggests overall similar placement characteristics and flow rates between the BPIO and SPIO when used to place IO catheters in dogs and cats. Results of this study should be interpreted cautiously due to small numbers and lower than previously reported success in IO catheter placement. This is especially true for our low success rate in cats. Further studies are needed to continue to explore the differences between both devices, training requirements, and clinical applications.

## Data availability statement

The raw data supporting the conclusions of this article will be made available by the authors, without undue reservation.

## Ethics statement

Ethical review and approval was not required for the animal study because the study was performed on cadavers.

## Author contributions

LG and JG designed the study, collected and analyzed data, co-wrote the manuscript and equally contributed as senior authors. OU collected and analyzed data and co-wrote the manuscript. KH assisted in study design and provided edits to the manuscript. CT assisted in collecting the data. TW and JD assisted in study design and provided edits to the manuscript. All authors contributed to the article and approved the submitted version.

## Funding

The authors declare that this study received funding from SAM Medical Inc. The funder was not involved in the study design, collection, analysis, interpretation of data, the writing of this article or the decision to submit it for publication.

## Conflict of interest

LG and JG are reviewers for the Journal, but only participated in this peer review process as researchers.

The remaining authors declare that the research was conducted in the absence of any commercial or financial relationships that could be construed as a potential conflict of interest.

## Publisher’s note

All claims expressed in this article are solely those of the authors and do not necessarily represent those of their affiliated organizations, or those of the publisher, the editors and the reviewers. Any product that may be evaluated in this article, or claim that may be made by its manufacturer, is not guaranteed or endorsed by the publisher.

## Footnotes

^1^Arrow EZIO, Teleflex, Wayne, PA.

^2^SAM IO, SAM-medical, Tualatin, OR.

^3^EZIO technique – https://www.youtube.com/watch?v=10twNYP1pB0.

^4^SAM IO technique – https://www.youtube.com/watch?v=o7n5G3KXKD0.

^5^Skulls Unlimited International Inc., Oklahoma City, OK.

^6^eNasco, Fort Atkinson, WI.

^7^0.9% NaCl, ICU Medical Inc., Lake Forest, IL.

^8^Infusion set Back check valve, BD Alaris, Park Terre-Bonne, Switzerland.

^9^Scale, Amazon Basics, Amazon Corporate Headquarters; Seattle, WA.

^10^Pressure sleeve, Medex Inc., Hilliard, OH.

^11^OEC Elite Model 5,455,288–03, GE HealthCare, Chicago, IL.

^12^Iohexol, NovaPlus, Vizient, Irving, TX.

^13^SAS v9.4, SAS Institute Inc., Cary, NC.

## Abbreviations

IO: intraosseous
BPIO: battery-powered intraosseous device
SPIO: spring-powered intraosseous device
IV: Intravenous
